# Status of pediatric echocardiography clinical trials: a cross-sectional study of registered trials in ClinicalTrials.gov

**DOI:** 10.3389/fped.2023.1167278

**Published:** 2023-04-25

**Authors:** Li-Juan Mao, Lan Wang, Dong-Mei Lv

**Affiliations:** ^1^ Department of Pediatric Ultrasound, The First Hospital of Jilin University, Changchun, China; ^2^ Department of Ultrasound Medicine, The Second Hospital of Jilin University, Changchun, China

**Keywords:** clinical trial, echocardiography, pediatric, ClinicalTrials.gov, imaging

## Abstract

**Background:**

The objective of this study is to analyze the characteristics of pediatric echocardiography clinical trials registered in ClinicalTrials.gov.

**Methods:**

A data set including pediatric echocardiography clinical trials was downloaded from ClinicalTrials.gov until May 13, 2022. We searched the PubMed, Medline, Google Scholar, and Embase databases to extract publication data. Pediatric echocardiography trial characteristics, application areas, and publication status were described. The secondary objectives were to evaluate factors associated with trial publication.

**Results:**

We identified 410 pediatric echocardiography reporting definite age, of which 246 were interventional and 146 were observational. Drug interventions were the most commonly studied (32.9%). The most applied area of pediatric echocardiography was congenital heart disease, followed by hemodynamics of preterm or neonatal infants, cardiomyopathy, inflammatory heart disease, pulmonary hypertension, and cardio-oncology. According to the primary completion data, 54.9% of the trials were completed before August 2020. 34.2% of the trials had been published within 24 months. Union countries and quadruple masking were more likely to be published.

**Conclusion:**

Echocardiography is rapidly evolving in pediatric clinical applications, including anatomic imaging and functional imaging. Novel speckle tracking techniques have also been pivotal in the assessment of cancer therapeutics-related cardiac dysfunction. A small number of clinical trials in pediatric echocardiography are published in a timely fashion. Concerted efforts are needed to promote trial transparency.

## 1. Introduction

Adequate methodological quality and complete, credible reporting in clinical trials are essential for medical development and innovation (1, 2). The ClinicalTrials.gov Web site was established by the Department of Health and Human Services based on the 1997 Food and Drug Administration Modernization Act and is maintained by the National Library of Medicine at the National Institute of Health. It aims to broaden patient access to novel drugs and medical equipment, improve research transparency, and reduce selective publication of research studies (3–5). In 2007, Section 801 of the Food and Drug Administration Amendments Act (FDAAA) expanded this mandate by requiring sponsors of applicable clinical trials to register and report basic summary results at ClinicalTrials.gov. Currently, ClinicalTrials.gov is the largest and most extensive platform for clinical trial registration studies in the world (6). However, the homogeneity of the pediatric population, the general underrepresentation of children in clinical research, and the limited pediatric evidence base have resulted in children being an underserved population in clinical research (7, 8).

According to statistics, a total of 3.12 million babies were born with congenital heart anomalies in 2019, representing 2305.2 per 100 000 live births (9). With advances in diagnostic methods and improvements in surgical procedures, the survival rate of patients with congenital heart disease has further improved. In 2019, the age-adjusted death rate (deaths per 100 100 people) attributable to congenital heart disease was 0.9, an 18.2% decrease from 2009 (10). Besides, From NHANES data, the overall prevalence of obesity in youth 2 to 19 years of age increased from 13.9% to 19.3% between 1999 and 2000 and from 2017 to 2018 (11). Thus, childhood congenital heart disease and acquired cardiovascular diseases, such as hypertension, obesity, and cardio-oncology, are associated with significant morbidity and mortality, as well as a high societal healthcare burden (12).

Many cardiovascular diseases begin in early childhood, which provides a new opportunity for prevention and guided decision-making. Echocardiography is the first imaging technique to screen, diagnose, and monitor pediatric heart disease. Given the paucity of data regarding the characteristics of pediatric echocardiography clinical trials, this study aimed to provide a comprehensive analysis of clinical trials in pediatric echocardiography registered with ClinicalTrials.gov to elucidate their special features. Furthermore, we further explored the utility of echocardiography and its novel techniques in pediatric congenital and acquired heart disease and assessed factors associated with trial publication. These contribute to the implementation of high-quality pediatric echocardiography trials and advance the development of pediatric echocardiography.

## 2. Materials and methods

### 2.1. Inclusion criteria and exclusion criteria

The inclusion criteria were registered trials on pediatric echocardiography; The exclusion criteria were registered trials on adult echocardiography and incomplete age information.

### 2.2. Data search

We searched the ClinicalTrials.gov on May 13, 2022, for the pediatric echocardiography Clinical Trials. The trials were obtained from ClinicalTrials.gov using the advanced search function with the search term “echocardiography”, with eligibility criteria of Child (birth-18).

### 2.3. Data collection

As previously described, data on ClinicalTrials.gov are self-reported by trial sponsors or investigators using a Web-based system. We downloaded the raw search data in XML format for the entire registry from ClinicalTrials.gov. Two dedicated researchers independently screened the trials according to the inclusion and exclusion criteria. The full study record on ClinicalTrials.gov was examined if the obtained information needed to be identified. And then, two researchers independently extracted the data from the included trials. Disagreements, if any, were resolved either by consensus or by a third reviewer. All cross-sectional analyses were performed on data extracted from ClinicalTrials.gov, including the National Clinical Trials (NCT) number, study type, trial registration year, trial start year, enrollment, participant gender, age group of enrolled children, sponsor class, status, phase, trial report result, location, intervention model, masking, primary purpose, observational model, time perspective, and publication status.

### 2.4. Publication identification

According to the Food and Drug Administration Amendments Act (FDAAA), trial results should be reported by the sponsor within 1–2 years after the primary completion time. Therefore, trials with the primary completion time before May 13, 2020, or published at least two years before the search date were included. After identifying the included trials, we searched the PubMed, Embase, Web of Science, and Cochrane Library databases using the NCT number, investigator names, and study keywords. If multiple publications were retrieved, the earliest publication would be selected for further analysis.

### 2.5. Statistical analysis

A cross-sectional, descriptive study of clinical trials for pediatric echocardiography, which had been registered on the ClincialTrials.gov, was conducted. Standard summary statistics were created to describe trial characteristics. Categorical variables were presented as percentage frequencies. Continuous variables were presented as medians and ranges. Categorical variables were compared using the chi-test or Fisher's exact tests. Univariate and multivariate logistic regression analyses were used to identify the independent factors related to the publication of trial results. A stepwise variable selection was performed in the univariate analysis retaining all predictors with *P* values <0.05 in the multivariate analysis. The odds ratio (OR) and its associated 95% confidence interval (CI) were reported. We used the Kaplan-Meier method to calculate the cumulative publication percentage in the time since the primary completion time. Statistical analysis was performed using IBM SPSS Statistics software (version 22.0.0, IBM, NY). *P* values less than 0.05 were considered to indicate statistical significance.

## 3. Results

### 3.1. Basic trials characteristics

**Figure 1:**
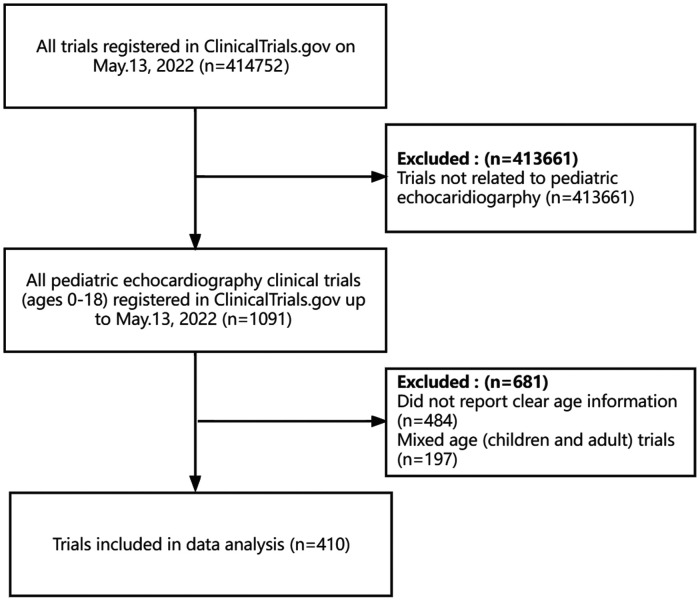
The flow chart of study clinical trials selection.

On May 13, 2022, we identified 1,091 pediatric echocardiography trials in the group of 414,752 registered in clinicalTrials.gov after the initial search, 681 (62%) of which were later excluded (Figure 1). The most common reason for exclusion was trials without clear age information (*n* = 484). We also excluded studies that were not related to echocardiography or that included some adults in addition to children (*n* = 197). Finally, a total of 410 trials were included in this study. Supplementary Table S1 showed clinical trial features and data classified by trial study type. A total of 246 trials were interventional, and 164 were observational. The largest proportion of trials (*n* = 179, 43.7%) had been completed, and 80 (19.5%) were still recruiting. The most common intervention category was drug intervention (*n* = 135, 32.9%), followed by the device (*n* = 43, 10.5%) and procedure (*n* = 31, 7.6%). 109 trials were open and not masking. Most trials (95.4%) recruited both male and female participants. With regard to age group, adolescent trials accounted for the largest proportion of trials (44.4% vs. 23.4% for the neonate trials). A similar trend existed in interventional and observational trials. Most trials were in a single country (*n* = 376, 91.7%), and the median (Q1, Q3) enrollment was 60 (30, 146.5) participants. The majority of trials began recruitment between 2018 and 2022, followed by 2013 and 2017. The same conditions were found for observational and interventional trials. For study duration, 406 trials (99%) had available information, and it ranged from less than 1 year to 17 years, with 72.0% having a duration of 1–5 years. The characteristics of the interventional and observational trials were similar, with more than two-thirds of trials lasting 1–5 years. For funding, 56 (13.7%) were funded by industries, and 323 (78.8%) did not report clear funding information. The characteristics of all 246 trials are summarized in Table 1. Some of the results reported in this table are highlighted here. More than half of the trials registered with ClincicalTrials.gov were randomized (*n* = 145, 58.9%), and ninety-seven (39.4%) trials were masked. For the types of assignments, most trials (*n* = 147, 59.8%) were parallel assignments, followed by single group assignments (33.3%). Treatment was the most frequently cited primary trial purpose, with a minority of trials tracking prevention (*n* = 28, 11.4%) and diagnostics (*n* = 19, 7.7%). More than half of the trials reported a phase date (*n* = 144, 58.5%). A small fraction of trials reported were phase 3 (*n* = 45, 18.3%), followed by phase 2. The characteristics of observational trials are displayed in Table 2. With respect to study design, the top 2 studies with a significant number of observational trials on ClinicalTrial.gov were cohort or case-control studies. The largest number of observational trials were prospective.

**Table 1:** The design of interventional studies.

Characteristic	Trials, *n* (%)
**Allocation**	
Randomized	145 (58.9%)
Non-randomized	29 (11.8%)
NA	72 (29.3%)
**Intervention model**	
Single group assignment	82 (33.3%)
Parallel assignment	147 (59.8%)
Crossover assignment	11 (4.5%)
Factorial assignment	2 (0.8%)
Sequential assignment	2 (0.8%)
NA	2 (0.8%)
**Masking**	
None (open label)	149 (60.6%)
Single	23 (9.3%)
Double	18 (7.3%)
Triple	15 (6.1%)
Quadruple	41 (16.7%)
**Primary purpose**	
Diagnostic	19 (7.7%)
Treatment	157 (63.8%)
Prevention	28 (11.4%)
Health services research	3 (1.2%)
Supportive care	7 (2.8%)
Screening	7 (2.8%)
Basic science	6 (2.4%)
Other	16 (6.5%)
NA	3 (1.2%)
**Phase**	
0 or 1	21 (8.5%)
1/2	18 (7.3%)
2	35 (14.2%)
2/3	10 (4.1%)
3	45 (18.3%)
4	15 (6.1%)
NA	102 (41.5%)

**Table 2:** The design of observational studies.

Characteristic	Trials, *n* (%)
**Observational model**	
Cohort	105 (64.0%)
Case-Only	19 (11.6%)
Case-Control	29 (17.7%)
Case-crossover	3 (1.8%)
Ecologic or Community	1 (0.6%)
Other	3 (1.8%)
NA	4 (2.4%)
**Time perspective**	
Prospective	125 (76.2%)
Retrospective	8 (4.9%)
Cross-Sectional	25 (15.2%)
Other	6 (3.7%)

### 3.2. Subclassifications by ultrasound type and topic

**Figure 2:**
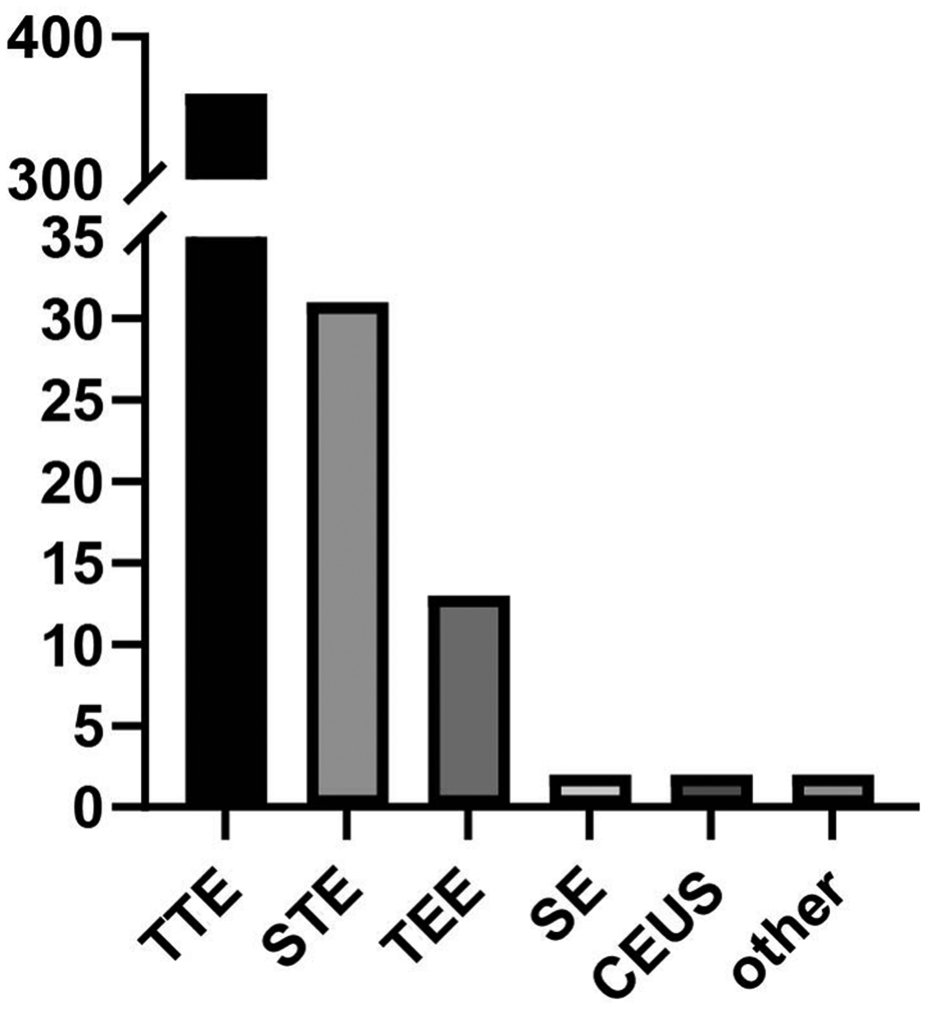
Clinical trial distribution with subclassifications by different echocardiography techniques.

Figure 2 depicts the clinical trial distribution with subclassifications by different echocardiography techniques. Multi-model echocardiography technology, including transthoracic echocardiography (TTE), speckle tracking echocardiography (STE), transesophageal echocardiography (TEE), stress echocardiography (SE), contrast-enhanced ultrasound (CEUS), pocket echocardiography and focused echocardiography. TTE trials were the most studies (*n* = 360, 87.8%), followed by STE (*n* = 31, 7.6%), TEE (*n* = 13, 3.2%), SE (*n* = 2, 0.5%), and CEUS (*n* = 2, 0.5%).

**Figure 3:**
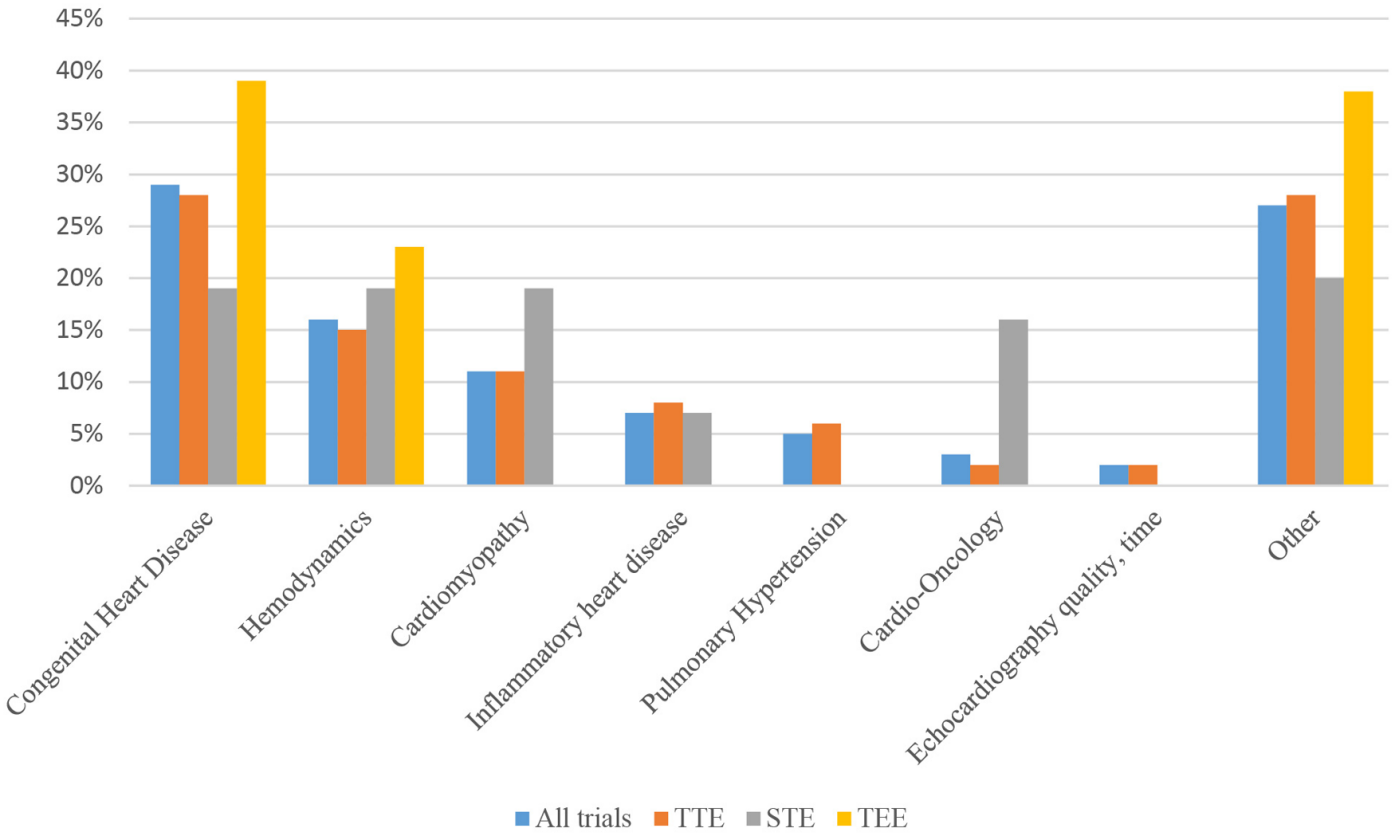
Comparison of application categories between different echocardiography techniques.

The primary application area of pediatric echocardiography clinical trials is displayed in Figure 3. The six conditions with the highest proportion were congenital heart disease, hemodynamics of preterm or neonatal infants, cardiomyopathy, inflammatory heart disease, pulmonary hypertension, and cardio-oncology. Specifically, inflammatory heart disease may be divided into Kawasaki disease, rheumatic heart disease, coronavirus disease 2019 (COVID-19), infective endocarditis, and pericarditis. Congenital heart disease was the most frequently studied disease category, accounting for 28.3% and 28.1% of all clinical trials and TTE trials, respectively. Other common topics for clinical trials included hemodynamic assessment (15.6% vs. 15.0%), cardiomyopathy (11.2 vs. 11.1%), and cardio-oncology (2.9% vs. 1.4%). Moreover, in regard to STE, we have detected 31 clinical trials, congenital heart disease (19.4%), hemodynamics (19.4%), and cardiomyopathy (19.4%) were the most common conditions among STE clinical trials, followed by cardio-oncology (16.1%). However, it was worth noting that the overall proportion of the cardio-oncology remained highest in STE clinical trials compared to all clinical trials and TTE trials. In terms of TEE clinical trials, congenital heart disease was the top one targeted condition. There is no significant difference in stratified analysis of interventional studies and observational studies.

### 3.3. Trial registration over time

**Figure 4:**
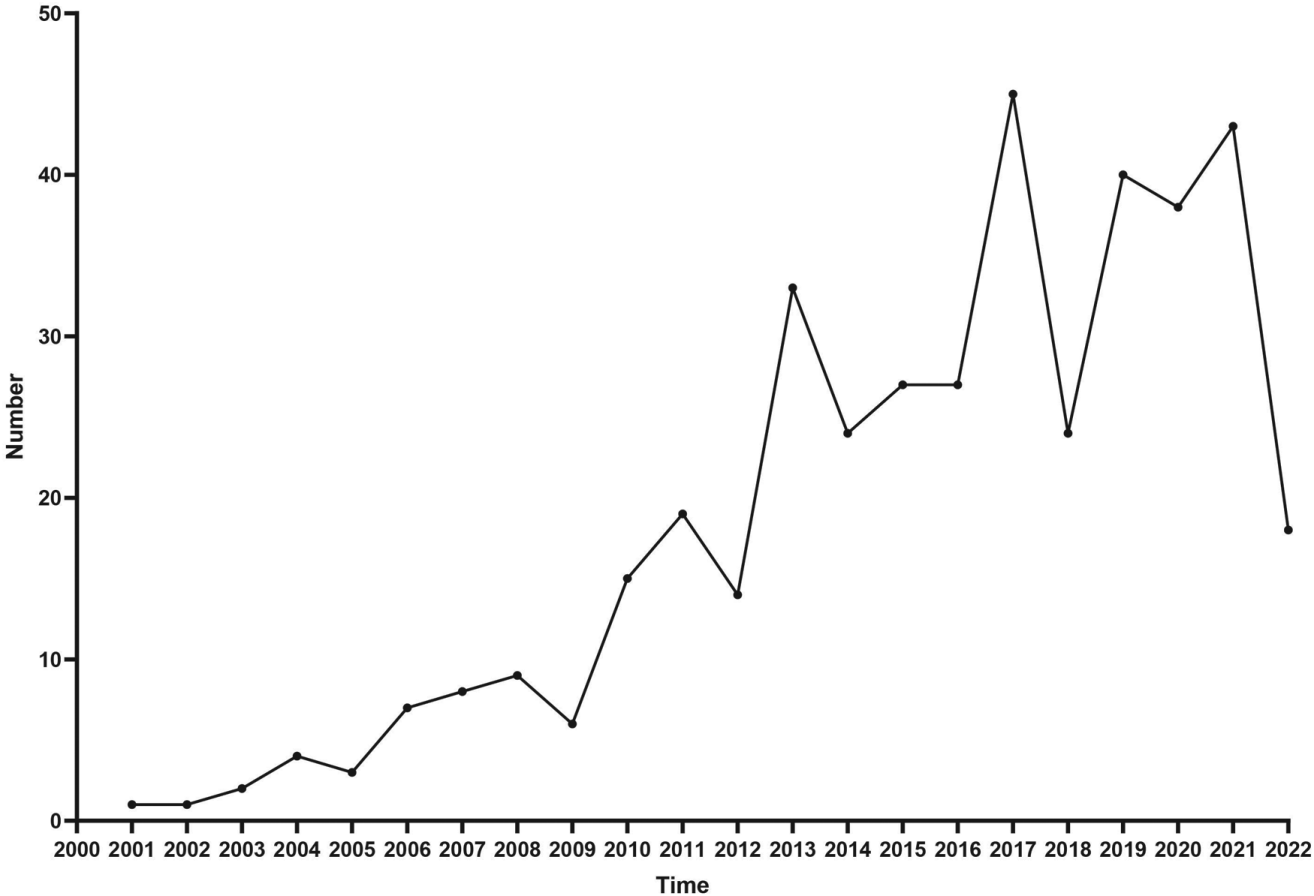
Number of trials on ClinicalTrials.gov by pediatric echocardiography per year.

The number of trial registrations per year is shown in Figure 4. There was an upward trend in the number of clinical trials as the years went go, with the highest number of newly registered trials in 2017 (*n* = 45). For reporting on clinical trials over time, of the 410 pediatric echocardiography clinical trials that we reviewed during the study period, according to the primary completion time, 225 (54.9%) trials were completed before August 2020.

### 3.4. Publication status and factors associated with the publication

**Figure 5:**
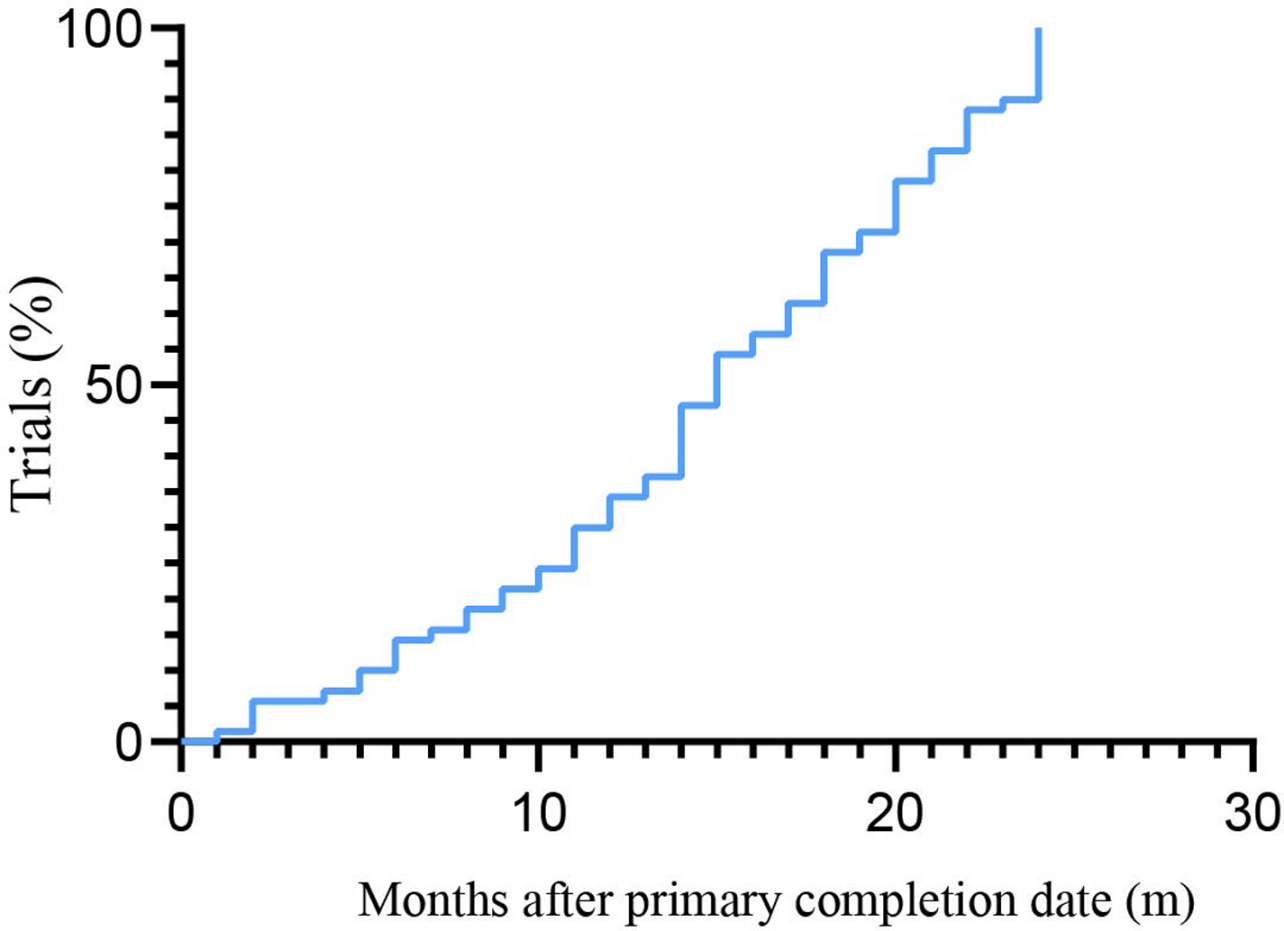
Cumulative publication percentage after the primary completion date.

Regarding the overall situation of publication status, among the 225 trials, 77 (34.2%) had been published within 24 months according to the primary completion time, presenting 23.3% (20/86) of the observational studies and 41.0% (57/139) of the interventional studies (Supplementary Table S2). Of the published trials, trials were categorized according to their interventional type, with drug studies comprising 42.9% of the trials, devices in 6.5%, biologics in 5.2%, and other (including diagnostic tests, procedures, and observational trials) in 45.5%. The largest funding source was industry (28.6%), followed by NIH (10.4%). The median time from primary completion time to publication was 13 (interquartile range, 8–20). Figure 5 displayed the cumulative percentage of publication after trial completion. The rate of publication was 24.3% at 10 months, 54.3% at 15 months, and 78.6% at 20 months.

The regression analyses of the unpublished and published trials are presented in Table 3. In univariate analysis, the factors that were associated with the publication by 24 months were trial phase (0.002), funding source (*P* < 0.001), study type (*P* = 0.006), location (*P* < 0.001), interventional model (*P* = 0.026), masking (*P* = 0.009), time perspective (*P* = 0.017), and completion year (*P* = 0.036). In multivariate regression analysis, the factors that were associated with the publication by 24 months included location and masking. As compared with a single country, an increase in publication for union countries was observed, with an adjusted hazard ratio of 41.93 (95% CI, 3.27 to 538, *P* = 0.004). Trials with quadruple masking were significantly more likely to be published than those that did not report masking, with an adjusted hazard ratio of 22.64 (95% CI, 1.83 to 280.37, *P* = 0.015).

**Table 3:** Multivariate analysis associated with publication for clinical trials.

Characteristic	Odds ratio (95% CI)	*P*
**Location**		
Single	1.00	
Union	41.93 (3.27–538)	0.004
**Masking**		
None (open label)	1.00	
Single	1.44 (0.27–7.67)	0.672
Double	3.06 (0.35–26.69)	0.311
Triple	0.9 (0.05–16.06)	0.940
Quadruple	22.64 (1.83–280.37)	0.015
NA	0 (0∼Inf)	0.782

## 4. Discussion

As cardiovascular disease continues to get younger, early cardiovascular disease prevention can generate a huge return on investment. Echocardiography, which is a radiation-free, inexpensive, and widely available imaging technique, has been recommended as the preferred imaging tool for the diagnosis of cardiovascular disease. Our analysis provides a descriptive assessment of the pediatric echocardiography clinical trials landscape from 1999 to 2022 and a comparison with unpublished and published trials. Thus, this analysis presents a unique opportunity for pediatric echocardiography clinical trials and identifies areas of relative strength or weakness.

Similar to prior ClinicalTrials.gov analyses in the large pediatric trials (13), the industry exclusively funded only 13.7% of these trials. More than two-thirds of pediatric echocardiography trials did not report a clear funding source, and they may be funded by foundations, academic institutions, and medical centers. Perhaps most concerning, however, is that many of the echocardiography trials conducted in the pediatric population are relatively small studies enrolling < 100 subjects—substantially smaller than the number of overall pediatric clinical trials. There are several explanations for this finding. First, pediatric clinical trials are often challenging due to scientific, ethical, and practical factors; second, pediatric echocardiography, as a subclassification of pediatric clinical trials, focuses on rare congenital cardiac malformations, which is consistent with our later findings. Third, congenital heart disease often requires surgical intervention, which poses challenges for study design and clinical endpoints. A previous report showed there were 247 large, nonvaccine pediatric RCTs with ≥1,000 participants registered in ClinicalTrials.gov before January 2020, and only 17 pediatric mega-trials with ≥5,000 participants exist (14). This indicates large pediatric clinical trials need further attention. However, several challenges remain, including establishing better infrastructures at study sites and greater recognition by academic institutions of the importance of these clinical trials to the advancement of pediatric medical care (15).

In our study, TTE trials were the most frequently registered trials for the diagnosis of congenital heart disease. It is well known that echocardiography has been the mainstay of anatomic and physiological assessment in pediatric cardiology and congenital heart disease. The American College of Cardiology (ACC), American Academy of Pediatrics (AAP), and American Heart Association (AHA) combined task force also published pediatric noninvasive cardiac imaging training guidelines in 2005, including guidelines for training in TTE, TEE et al. (16). With the advancement of echocardiography technique, new imaging methods such as strain imaging, CEUS, pocket echocardiography, and focused echocardiography have become more commonplace in everyday practice. Furthermore, this report suggests that other pediatric specialties with a relatively large volume included the following: hemodynamics of neonates and children, cardiomyopathy, inflammatory heart disease, and pulmonary hypertension. Currently, the role of echocardiography in the neonatal intensive care unit has changed from the diagnosis of congenital heart disease to the evaluation of hemodynamic instability. The terms functional echocardiography and point-of-care echocardiography have been introduced to describe the use of echocardiography as an adjunct in the clinical assessment of the hemodynamic status in neonates and children (17, 18). Just because preterm or neonatal infants are unique in that they are in the process of transition from fetal to postnatal circulation, and a large number of relevant clinical trials have followed. In 2015, Hill, Kevin D. et al. (19) reported that pediatric cardiovascular trials rarely focused on specific congenital malformations and more commonly focused on diseases and conditions considered to be high-impact adult cardiovascular diseases, including hypertension and pulmonary hypertension. However, in our study, pediatric echocardiography clinical trials emphasize the assessment of congenital heart disease; this may be indicated by the inherent advantages of echocardiography in anatomic identification. Surprisingly, for the echocardiography subclassification, cardio-oncology accounts for a significant proportion of STE-associated clinical trials. This finding deserves further in-depth consideration. The data shows that cancer is diagnosed in >12,000 children and adolescents in the United States annually. Progress in cancer therapeutics over the past years has remarkably improved survival rates for most children with malignancies. For all pediatric cancers, 5-year survival approached 85% for children diagnosed between 1999 and 2006 (20, 21). As childhood cancer survivors increased, cancer therapeutics-related cardiac dysfunction (CTRCD) became the most common noncancer cause of death among long-term childhood cancer survivors (22). Early identification of CTRCD is essential for childhood cancer survivors. STE plays an important role in the evaluation of cardiotoxicity related to tumor radiotherapy and chemotherapy because of its noninvasive and convenient advantages. Further clinical trials in pediatric echocardiography should make efforts to explore STE application in Cardio-Oncology. It is helpful to identify subclinical myocardial dysfunction associated with tumor radiotherapy and chemotherapy and guide clinical decision-making. Recently, a large meta-analysis revealed that left ventricular systolic deformation was impaired in children with cancer during the initial treatment phase and among long-term childhood cancer survivors (23). These findings indicated that further clinical trials were required to better understand strain parameters in the risk stratification of children with cancer. Advanced echocardiography techniques have provided precise diagnostic and prognostic benefits, and our study showed a significant increasing trend.

The FDA Amendments Act (FDAAA), enacted in September 2007 in the US, included new initiatives to use ClinicalTrials.gov to further address selective publication. The legislation requires that the results of clinical trials should be reported within 12 to 24 months. We also identified several findings. A small number of pediatric echocardiography have already been published in the peer-reviewed literature. At 24 months, results had been published for 34.2% of trials before August 2020. For interventional trials, the rate of publication was close to 41%. In 2015, Monique L et al. identified 13,327 clinical trials, and only 13.4% of trials reported summary results within 12 months after trial completion (24). In a recent pediatric clinical trial study, the results showed that 88.5% of registered studies (from January 2008 to December 2019) did not report results (25). Publication bias is a well-known phenomenon; that is, trials with positive findings are published more quickly and more frequently. Clinical trial registration is an important step for helping protect against publication bias and increasing clinical trial transparency (26, 27). Selective reporting of studies means that fully informed decisions cannot be made about the care of patients, resource allocation, prioritization of research questions, or study design. To ensure transparency, the results database should strengthen the practice of systematic reporting, thereby facilitating communications between the public and the profession.

Multivariable analysis found that union country was associated with publication within 24 months after trial completion, but single was not. Several factors may help to explain the difference in publication rate; an essential factor in this discrepancy may be publication bias, as basic results are required to report for clinical trials funded by all National Institutes of Health (HIH) (28). Trials from multiple countries may have adequate patient accrual, funding source, and are more likely to report results (29, 30). Furthermore, international trials are more powerful and credible, with relatively satisfactory recruitment. Quadruple masking was also associated with the timely disclosure of trial publication results. This result may be due to the fact that the quadruple masking helps to eliminate or at least minimize some potential bias in clinical trials. Another study also showed quadruple masking was associated with increased publication rates (31). Their results are akin to our findings.

## 5. Limitations

There are several limitations that should be noted. First, there are some trials that are not registered in ClinicalTrials.gov but are registered in other clinical trials registries, such as the China Clinical Trials Registry and the World Health Organization's International Clinical Trials Registry, and these studies are not included in our evaluation. In particular, the current FDAAA does not require some trials to be registered in ClinicalTrials.gov, including Phase I investigations, small clinical trials to determine feasibility, and specific clinical trials to test prototype devices. Still, ClinicalTrials.gov accounts for more than 70% of all clinical studies in the World Health Organization International Clinical Trials Registry Platform. Second, there is no standard or comprehensive classification for echocardiography in pediatric diseases, we categorized these trials by manually coding keywords, so our analysis is still susceptible to selection bias. Third, we did not evaluate and compare certain parameters of trials from published articles, such as the results and conclusions of pediatric echocardiography trials. Therefore, future studies could attempt to elucidate these details. In addition, there is a significant amount of missing or unsubmitted data for certain data fields, which limits the comprehensiveness of the analysis that can be performed with these data.

## 6. Conclusion

In conclusion, analysis of the ClinicalTrials.gov data set al.lows a description of the current status of pediatric echocardiography trials. Our study utilized this publicly available resource to gain insight into the recent advances in pediatric echocardiography, clinical practice, and factors related to the trial publication. Our results showed that application of the echocardiography in different pediatric cardiovascular diseases was unbalanced. The publication rate of pediatric echocardiography trials remained low and was associated with location and masking. Echocardiography is a valuable noninvasive tool in screening, diagnosing, and assessing pediatric diseases. The pediatric echocardiography community is small but cohesive. Our study can contribute to perfecting experimental design and conducting the optimal clinical trials to improve the care for children with cardiovascular diseases.

## Data Availability

Publicly available datasets were analyzed in this study. This data can be found here: https://clinicaltrials.gov/.
