# Supplementary material for: Status of pediatric echocardiography clinical trials: a cross-sectional study of registered trials in ClinicalTrials.gov
Source: Front Pediatr. 2023 Apr 25;11:1167278. doi: 10.3389/fped.2023.1167278 (PMC10167035; doi:10.3389/fped.2023.1167278)
Supplement: Supplementary file 1 [file Table1.docx]

Supplementary Material

Status of pediatric echocardiography Clinical Trials:

A Cross-Sectional Study of Registered Trials in ClinicalTrials.gov

Li-Juan Mao ^1*, †^, Lan Wang ^2,^ ^†^, Dong-Mei LV ^3^

*** Correspondence:** Corresponding Author: maolijuan1026@163.com.

# Supplementary Tables

## Supplementary Table 1. Characteristics of pediatric echocardiography trials registered before May 13, 2022

| **Characteristics** | | All trials  No. (%)  (n = 410) | | Observational trials No. (%)  (n = 164) | Interventional trials No. (%)  (n = 246) |
| --- | --- | --- | --- | --- | --- |
| **Status** | | | | |  |
| Completed | 179 (43.7%) | | 76 (46.3%) | 103 (41.9%) |  |
| Recruiting | 80 (19.5%) | | 35 (21.3%) | 45 (18.3%) |  |
| Unknown | 59 (14.4%) | | 22 (13.4%) | 37 (15%) |  |
| Not yet recruiting | 29 (7.1%) | | 14 (8.5%) | 15 (6.1%) |  |
| Withdrawn | 7 (1.7%) | | 2 (1.2%) | 5 (2%) |  |
| Active, not recruiting | 24 (5.9%) | | 8 (4.9%) | 16 (6.5%) |  |
| Enrolling by invitation | 9 (2.2%) | | 4 (2.4%) | 5 (2%) |  |
| Terminated | 22 (5.4%) | | 3 (1.8%) | 19 (7.7%) |  |
| Suspended | 1 (0.2%) | | 0 (0%) | 1 (0.4%) |  |
| **Open** | | | | |  |
| No | 301 (73.4%) | | 115 (70.1%) | 186 (75.6%) |  |
| Yes | 109 (26.6%) | | 49 (29.9%) | 60 (24.4%) |  |
| **Type** | | | | |  |
| Drug | 135 (32.9%) | | 8 (4.9%) | 127 (51.6%) |  |
| Procedure | 31 (7.6%) | | 6 (3.7%) | 25 (10.2%) |  |
| Device | 43 (10.5%) | | 30 (18.3%) | 13 (5.3%) |  |
| Diagnostic test | 31 (7.6%) | | 21 (12.8%) | 10 (4.1%) |  |
| Biological | 20 (4.9%) | | 0 (0%) | 20 (8.1%) |  |
| Behavioral | 13 (3.2%) | | 1 (0.6%) | 12 (4.9%) |  |
| Others | 76 (18.5%) | | 37 (22.6%) | 39 (15.9%) |  |
| Not available | 61 (14.9%) | | 61 (37.2%) | 0 (0%) |  |
| **Gender** | | | | |  |
| All | 391 (95.4%) | | 158 (96.3%) | 233 (94.7%) |  |
| Female | 1 (0.2%) | | 1 (0.6%) | 0 (0%) |  |
| Male | 18 (4.4%) | | 5 (3%) | 13 (5.3%) |  |
| **Age-group** | | | | |  |
| Neonates, 0–30 d | 96 (23.4%) | | 37 (22.6%) | 59 (24%) |  |
| Infants, 30 d–1 y | 62 (15.1%) | | 18 (11%) | 44 (17.9%) |  |
| Children, 1–12 y | 70 (17.1%) | | 28 (17.1%) | 42 (17.1%) |  |
| Adolescents, 12–18 y | 182 (44.4%) | | 81 (49.4%) | 101 (41.1%) |  |
| **Enrollment (No. of patients)** | |  | | |  |
| 0-50 | 164 (40.0%) | | 47 (28.7%) | 117 (47.6%) |  |
| 50-100 | 86 (21.0%) | | 37 (22.6%) | 49 (19.9%) |  |
| 100-500 | 126 (30.7%) | | 56 (34.1%) | 70 (28.5%) |  |
| 500-1000 | 11 (2.7%) | | 6 (3.7%) | 5 (2%) |  |
| >1000 | 23 (5.6%) | | 18 (11%) | 5 (2%) |  |
| **Trial start year** | | | | |  |
| Before 2007 | 19 (4.6%) | | 6 (3.7%) | 13 (5.3%) |  |
| 2007-2012 | 57 (13.9%) | | 22 (13.4%) | 35 (14.2%) |  |
| 2013-2017 | 125 (30.6%) | | 45 (27.4%) | 80 (32.5%) |  |
| 2018-2022 | 208 (50.9%) | | 91 (55.4%) | 117 (47.6%) |  |
| NA | 1 (0.2%) | | 0 (0%) | 1 (0.4%) |  |
| **Study duration (y)** | | | | |  |
| 0-1 | 63 (15.4%) | | 32 (19.5%) | 31 (12.6%) |  |
| 1-5 | 295 (72.0%) | | 111 (67.7%) | 184 (74.8%) |  |
| 5-10 | 43 (10.5%) | | 17 (10.4%) | 26 (10.6%) |  |
| >10 | 5 (1.2%) | | 4 (2.4%) | 1 (0.4%) |  |
| NA | 4(1.0%) | | 0 (0%) | 4 (1.6%) |  |
| **Location** | | | | |  |
| Union | 34 (8.3%) | | 2 (1.2%) | 32 (13%) |  |
| Single | 376 (91.7%) | | 162 (98.8%) | 214 (87%) |  |
| **Funded-by** | | | | |  |
| Industry | 56 (13.7%) | | 4 (2.4%) | 52 (21.1%) |  |
| NIH | 25 (6.1%) | | 12 (7.3%) | 13 (5.3%) |  |
| Industry+ NIH | 4 (1.0%) | | 1 (0.6%) | 3 (1.2%) |  |
| US. Fed | 2 (0.5%) | | 1 (0.6%) | 1 (0.4%) |  |
| Other | 323 (78.8%) | | 146 (89%) | 177 (72%) |  |

## Supplementary Table 2. Comparison of published and unpublished trials completed before May 13, 2022

| **Characteristic** | Total  No. (%)  (n = 225) | | Unpublished Studies No. (%)  (n = 148) | Published Studies  No. (%)  (n = 77) | P-value |
| --- | --- | --- | --- | --- | --- |
| **Type** | |  |  |  |  |
| Drug | | 81 (36.0%) | 48 (32.4%) | 33 (42.9%) | 0.148 |
| Biological | | 7 (3.1%) | 3 (2%) | 4 (5.2%) |  |
| Device | | 23 (10.2%) | 18 (12.2%) | 5 (6.5%) |  |
| Other | | 114 (50.7%) | 79 (53.4%) | 35 (45.5%) |  |
| **Gender** | |  |  |  |  |
| All | | 214 (95.1%) | 143 (96.6%) | 71 (92.2%) | 0.192 |
| Male | | 11 (4.9%) | 5 (3.4%) | 6 (7.8%) |  |
| **Age-group** | | |  |  |  |
| Neonates,0–30 d | | 55 (24.4%) | 43 (29.1%) | 12 (15.6%) | 0.074 |
| Infants,30 d–1 y | | 31 (13.8%) | 19 (12.8%) | 12 (15.6%) |  |
| Children,1–12 y | | 43 (19.1%) | 23 (15.5%) | 20 (26%) |  |
| Adolescents,12–18y | | 96 (42.7%) | 63 (42.6%) | 33 (42.9%) |  |
| **Phases** | | |  |  |  |
| Early Phase 1 | | 1 (0.4%) | 1 (0.7%) | 0 (0%) | 0.002 |
| Phase 1 | | 8 (3.6%) | 7 (4.7%) | 1 (1.3%) |  |
| Phase 1\|Phase 2 | | 10 (4.4%) | 8 (5.4%) | 2 (2.6%) |  |
| Phase 2 | | 19 (8.4%) | 10 (6.8%) | 9 (11.7%) |  |
| Phase 2\|Phase 3 | | 5 (2.2%) | 1 (0.7%) | 4 (5.2%) |  |
| Phase 3 | | 25 (11.1%) | 11 (7.4%) | 14 (18.2%) |  |
| Phase 4 | | 10 (4.4%) | 9 (6.1%) | 1 (1.3%) |  |
| NA | | 61 (27.1%) | 35 (23.6%) | 26 (33.8%) |  |
| Missing | | 86 (38.2%) | 66 (44.6%) | 20 (26%) |  |
| **Enrollment** | | |  |  | 0.103 |
| 0-100 | | 157 (69.8%) | 110 (74.3%) | 47 (61%) |  |
| 100-1000 | | 54 (24.0%) | 31 (20.9%) | 23 (29.9%) |  |
| >1000 | | 14 (6.2%) | 7 (4.7%) | 7 (9.1%) |  |
| **Funded-by** | | |  |  | < 0.001 |
| Industry | | 35 (15.6%) | 13 (8.8%) | 22 (28.6%) |  |
| NIH | | 15 (6.7%) | 7 (4.7%) | 8 (10.4%) |  |
| Other | | 175 (77.8%) | 128 (86.5%) | 47 (61%) |  |
| **Study type** | | |  |  | 0.006 |
| Observational | | 86 (38.2%) | 66 (44.6%) | 20 (26%) |  |
| Interventional | | 139 (61.8%) | 82 (55.4%) | 57 (74%) |  |
| **Location** | | |  |  | < 0.001 |
| Union | | 21 (9.3%) | 3 (2%) | 18 (23.4%) |  |
| Single | | 204 (90.7%) | 145 (98%) | 59 (76.6%) |  |
| **Allocation** | | |  |  | 0.399 |
| NA | | 125 (55.6%) | 85 (57.4%) | 40 (51.9%) |  |
| Non-randomized | | 16 (7.1%) | 12 (8.1%) | 4 (5.2%) |  |
| Randomized | | 84 (37.3%) | 51 (34.5%) | 33 (42.9%) |  |
| **Intervention Model** | | | |  | 0.026 |
| Single group assignment | | 46 (20.4%) | 24 (16.2%) | 22 (28.6%) |  |
| Parallel assignment | | 86 (38.2%) | 53 (35.8%) | 33 (42.9%) |  |
| Crossover assignment | | 4 (1.8%) | 4 (2.7%) | 0 (0%) |  |
| Factorial assignment | | 1 (0.4%) | 1 (0.7%) | 0 (0%) |  |
| NA | | 88 (39.1%) | 66 (44.6%) | 22 (28.6%) |  |
| **Masking** | | |  |  | 0.009 |
| None (open label) | | 81 (36.0%) | 52 (35.1%) | 29 (37.7%) |  |
| Single | | 16 (7.1%) | 10 (6.8%) | 6 (7.8%) |  |
| Double | | 11 (4.9%) | 8 (5.4%) | 3 (3.9%) |  |
| Triple | | 7 (3.1%) | 5 (3.4%) | 2 (2.6%) |  |
| Quadruple | | 22 (9.8%) | 7 (4.7%) | 15 (19.5%) |  |
| NA | | 88 (39.1%) | 66 (44.6%) | 22 (28.6%) |  |
| **Primary Purpose** | | | |  | 0.106 |
| Diagnostic | | 9 (4.0%) | 5 (3.4%) | 4 (5.2%) |  |
| Treatment | | 85 (37.8%) | 54 (36.5%) | 31 (40.3%) |  |
| Prevention | | 15 (6.7%) | 6 (4.1%) | 9 (11.7%) |  |
| Other | | 25 (11.1%) | 16 (10.8%) | 9 (11.7%) |  |
| NA | | 91 (40.4%) | 67 (45.3%) | 24 (31.2%) |  |
| **Observational Model** | | | |  | 0.121 |
| Cohort | | 47 (20.9%) | 37 (25%) | 10 (13%) |  |
| Case-Only | | 13 (5.8%) | 10 (6.8%) | 3 (3.9%) |  |
| Case Control | | 16 (7.1%) | 10 (6.8%) | 6 (7.8%) |  |
| Case-crossover | | 2 (0.9%) | 2 (1.4%) | 0 (0%) |  |
| Ecologic or Community | | 1 (0.4%) | 1 (0.7%) | 0 (0%) |  |
| Other | | 3 (1.3%) | 3 (2%) | 0 (0%) |  |
| NA | | 143 (63.6%) | 85 (57.4%) | 58 (75.3%) |  |
| **Time Perspective** | | | |  | 0.017 |
| Retrospective | | 3 (1.3%) | 2 (1.4%) | 1 (1.3%) |  |
| Cross-Sectional | | 15 (6.7%) | 9 (6.1%) | 6 (7.8%) |  |
| Prospective | | 65 (28.9%) | 53 (35.8%) | 12 (15.6%) |  |
| Other | | 3 (1.3%) | 2 (1.4%) | 1 (1.3%) |  |
| NA | | 139 (61.8%) | 82 (55.4%) | 57 (74%) |  |
| **Completion Year** | | |  |  | 0.036 |
| 1999 | | 1 (0.4%) | 1 (0.7%) | 0 (0%) |  |
| 2003 | | 1 (0.4%) | 1 (0.7%) | 0 (0%) |  |
| 2004 | | 1 (0.4%) | 1 (0.7%) | 0 (0%) |  |
| 2005 | | 1 (0.4%) | 1 (0.7%) | 0 (0%) |  |
| 2006 | | 3 (1.3%) | 3 (2%) | 0 (0%) |  |
| 2007 | | 2 (0.9%) | 1 (0.7%) | 1 (1.3%) |  |
| 2008 | | 6 (2.7%) | 4 (2.7%) | 2 (2.6%) |  |
| 2009 | | 3 (1.3%) | 2 (1.4%) | 1 (1.3%) |  |
| 2010 | | 6 (2.7%) | 5 (3.4%) | 1 (1.3%) |  |
| 2011 | | 13 (5.8%) | 11 (7.4%) | 2 (2.6) |  |
| 2012 | | 9 (4.0%) | 8 (5.4%) | 1 (1.3) |  |
| 2013 | | 21 (9.3%) | 14 (9.5%) | 7 (9.1) |  |
| 2014 | | 16 (7.1%) | 11 (7.4%) | 5 (6.5) |  |
| 2015 | | 19 (8.4%) | 18 (12.2%) | 1 (1.3) |  |
| 2016 | | 26 (11.6%) | 17 (11.5%) | 9 (11.7) |  |
| 2017 | | 26 (11.6%) | 15 (10.1%) | 11 (14.3) |  |
| 2018 | | 32 (14.2%) | 16 (10.8%) | 16 (20.8) |  |
| 2019 | | 32 (14.2%) | 17 (11.5%) | 15 (19.5) |  |
| 2020 | | 7 (3.1%) | 2 (1.4%) | 5 (6.5) |  |
